# Pasting and Dough Rheological Properties of Ackee (*Blighia sapida*) Aril Flour: A Contribution to the Search for Wheat Flour Substitutes

**DOI:** 10.1155/2021/5526912

**Published:** 2021-04-24

**Authors:** Ramiro Torres-Gallo, Ricardo Durán, José García-Camargo, Oswaldo Morales, Diofanor Acevedo, Diego F. Tirado

**Affiliations:** ^1^Department of Agroindustrial Engineering, School of Engineering, Universidad del Atlántico, km 07, Road to Puerto Colombia, 232527 Barranquilla, Colombia; ^2^Grupo de Optimización Agroindustrial (GOA), Universidad Popular del Cesar, Campus Sabanas, 200003, Sabanas del Valle, Valledupar, Colombia; ^3^Grupo de Investigación en Innovación y Desarrollo Agropecuario y Agroindustrial (IDAA), Universidad de Cartagena, Campus Piedra de Bolívar, Av. Consulado, # 48-152, 130015 Cartagena de Indias, Colombia

## Abstract

Wheat is one of the most widely used cereals in the world. However, studies consider wheat flour doughs to be of low nutritional quality, as there is now greater public awareness of celiac disease and gluten intolerance. Therefore, consumers are demanding healthier and more varied food products. Consequently, wheat flour is being replaced fully or partially by flours from other sources with higher quality. Hence, the main objective of this work was to report the effect of blending wheat flour with ackee aril flour, until the total replacement of wheat flour, on pasting and dough rheological properties. Five different levels of blending were analyzed: wheat to ackee aril flour mass ratios of 100 : 0, 75 : 25, 50 : 50, 25 : 75, and 0 : 100. Pasting properties (pasting temperature, peak viscosity, ease of cooking, swelling power, final viscosity at 50  °C, and thixotropy) were analyzed; and steady-state shear measurements were used to obtain consistency coefficients (*K*) and flow behavior indexes (*n*) after data was fitted to the Power Law and Herschel-Bulkley models. The gradual addition of the ackee aril flour fraction produced an increase in ash, fat, protein, and fiber content; while water and carbohydrate content showed the opposite behavior in the obtained composite flour. Consequently, the partial or full replacement of wheat flour changed the rheological properties of the produced doughs, as well as the quality of the final product. These changes were mostly related to the protein and carbohydrate content of the ackee aril flour fraction. In general, doughs showed a pseudoplastic behavior with thixotropy whose viscosity decreased as the addition of ackee aril flour was increased. Pasting properties of blends involving 25 %-75 % ackee aril flour demonstrate the feasibility of including these flours in products subjected to high processing temperatures such as canned products or even to produce chips and pasta.

## 1. Introduction

Wheat is one of the most widely used cereals for flour production [1]. Wheat flour use has spread all over the world because of its gluten fraction, which is responsible for the elasticity of the dough [1]. However, it is well known that wheat flour doughs are considered to be of low nutritional quality, as there is now greater public awareness of celiac disease and gluten intolerance [2], as well as consumer demands for healthy food and variety in food products [3].

On the other hand, wheat is not a major crop in Caribbean countries such as Colombia; so, wheat flour importation becomes mandatory to meet the local demand [4]. Therefore, the partial or full replacement of wheat flour by flours with indigenous origins and better nutritional profiles would undoubtedly improve the nutritional quality of the products [5, 6], satisfying the current demand for a healthy diet and varied food products [7].

There is an interest in replacing common gluten-free formulations made from gluten-free refined flour, starch [8], and hydrocolloids [9] with others enriched with gluten-free functional ingredients [10]. However, it should be kept in mind that the replacement of wheat flour by flours from other raw materials will alter the rheological properties of the dough [11], as well as the quality of the final product [12].

Concerning the above, *Blighia sapida* is a tree native to West Africa that produces the ackee fruit [13]. The tree belongs to the *Sapindaceae* family; and its fruit consists of a pod, pulp, and aril covering the seed [14]. Arils of the fruit only become edible and perfectly safe when the ripe fruit opens spontaneously to reveal the seeds (which are known to be poisonous) and the fleshy aril [15]. These edible arils are a rich source of protein, fat, and vitamins A, B1, B2, and C [14], thus, being a potential gluten-free substitute for wheat [16].

Ackee is mainly consumed in Jamaica for use in the national dish: ackee and salted fish. For this, the fruit is usually boiled gently for up to half an hour. However, even though the ackee fruit is cheap, has high dietary protein content (over 20 %) and has some claimed disease preventive properties, it is underutilized, and the quantity consumed worldwide is currently relatively low [13]. One of the possible reasons for the low consumption worldwide is that it is well known that when ingested unripe, ackee produces vomiting and fatal cases of poisoning [17]. The authors of this study consider that this low ackee intake could be solved if new forms of consumption/use of the fruit are sought, giving it added value at the same time. As an example of this, Essuman et al. [18] produced chips from ackee aril flour. Despite all this, no basic engineering studies have been carried out on ackee-based products. In this regard, the study of rheological properties is important in Food Science [6], since its knowledge is useful to define quality attributes in food products; and its understanding is also advantageous for packaging and stability prediction of stored samples [19].

Various studies have been carried out to study the effect of varying flour proportions in wheat flour mixtures on dough rheological properties [11, 20, 21]. All those studies claimed that the partial or full replacement of wheat flour changed the rheological properties of the produced doughs, as well as the quality of the final product.

The substitution of wheat flour by other types of flour is important from the economic point of view in Colombia and Latin America, since wheat is mainly an imported product in these areas [4]. Moreover, wheat flour is considered to be of low nutritional quality [2]. In this regard, ackee aril flour has potential as a wheat flour substitute in these world zones since the fruit could be easily grown [4], is cheap, has a high dietary protein content [4, 13, 22], and claimed functional properties such as good solubility, swelling power, oil absorption capacity, foaming capacity, foam stability, emulsion capacity, and emulsion stability [23]. Taking into account all of the above and that to the best of our knowledge, neither the study of pasting properties nor the rheological behavior of flour dough from ackee arils have been reported, the main objective of this work was to analyze the effect of blending wheat flour with ackee aril flour, until the complete replacement of wheat flour on pasting and dough rheological properties, with the engineered purpose of enhancing the functional properties, product quality, and processing performance of the flour.

## 2. Materials and Methods

### 2.1. Plant Material, Fruit Harvest, and Aril Obtention

Ackee (*B. sapida*) trees of a single genotype are growing in a private farm located within the urban perimeter of the municipality of Fonseca, La Guajira (Colombia). According to Benkeblia [24], fruits were harvested when fully open from the same tree and during the same period (March). Afterwards, the fruit was identified and authenticated at the laboratory (Valledupar, Colombia). Immediately after harvest, arils were carefully removed from the seeds and husk by pulling on the soft red membrane. This separation was easily done, as the fruit was ripe, and the red membrane was very soft. After their separation, arils were packed into polystyrene boxes at 4 °C before further use.

### 2.2. Flour Production from Ackee Arils

Ackee arils were washed with water and blanched at 55 °C for 10 min. Afterwards, the vegetable material was dried at 60 °C for 72 h in an oven (Digiheat, J.P. Selecta, S.A., Colombia). During the procedure, it was found that dried aril samples were fat-rich, which made it slightly difficult to obtain a fine flour. Consequently, the dried sample was milled with a hammer mill into flour and defatted with hexane using a ratio flour/solvent of 1/10 (*w*/*v*) for 24 h. Then, the defatted flour was repeatedly washed with clean water to get rid of all excess hexane and dried again at 60 °C for 3 h. Afterwards, the dried flour was milled again and sieved through a metal sieve of 2 mm pore size. Finally, ackee aril flour was sealed in polythene bags in airtight containers and kept in a refrigerator at 4 °C until ready for use.

### 2.3. Flour Blends

Commercial wheat flour was collected from a local market in Valledupar (Cesar). Flour mixtures with wheat to ackee aril flour mass ratios of 100 : 0 (T1); 75 : 25 (T2); 50 : 50 (T3), 25 : 75 (T4), and 0 : 100 (T5) were prepared. The negative control sample was 100 % wheat flour (T1). Each sample was blended in a high-speed blender and then sieved through a metal sieve of 2 mm pore size.

### 2.4. Physicochemical Properties of Flours

Physicochemical analyses of wheat flour, ackee aril flour, and wheat-ackee aril flour mixtures were carried out based on the official methods of analysis approved by the AOAC [25]. Ackee aril samples were analyzed regarding moisture (method 925.10) in an oven at 103 °C until the weight did not vary more than 0.1 %; ash (method 923.03) by burning the organic content, leaving inorganic minerals in the oven at 500 °C -600 °C for 24 h; fat (method 999.13) by Soxhlet extraction; protein (method 990.03) by the Kjeldahl method; crude fiber (method 978.10) in five steps (acid digestion, washing, alkali digestion, washing, and defatting); and finally total carbohydrate content by difference.

### 2.5. Pasting Properties

Pasting properties of the samples were determined using a rheometer TA AR 1500® (TA Instruments, United States) with a standard profile as described earlier by Singh et al. [26], with modifications. For this, 5 g of flour was weighed directly in a sample canister, and 40 mL distilled water was added. A heating and cooling cycle was programmed starting at a constant value of 50  °C, heated to 95  °C at 12  °C min^−1^, and a shear rate of 2.67  s^−1^, then held at 95  °C during 150 s, and cooled this time to 50  °C at 12  °C min^−1^ and the same frequency sweep. Parameters recorded were pasting temperature, peak viscosity, ease of cooking, swelling power, final viscosity at 50 °C, and thixotropy.

### 2.6. Rheological Measurements of Dough

Steady-state shear measurements were carried out with a rheometer TA AR 1500 using a plate of 40 mm controlled by the TA Universal Analysis Version 5.2® software with triplicate measurements. Dough samples prepared as described previously were equilibrated at 25 °C for about 15 min. Then steady-state shear experiments were carried out in the shear rate ($\overset{\text{.}}{\gamma }$) range of 0.01 s^−1^–100 s^−1^ for 300 s in the controlled shear rate mode at 25 °C. The rheological behavior of doughs described by the flow curves was fitted to the Power Law and Herschel-Bulkley models represented by Equation (1) and Equation (2), respectively.

(1) $$\begin{array}{c} \tau =K{\dot{\gamma }}^{n}, \end{array}$$

(2) $$\begin{array}{c} \tau =\tau_{0}+K{\dot{\gamma }}^{n}, \end{array}$$

where *τ* is the shear stress (Pa), *τ*_0_ is the yield stress (Pa), $\overset{\text{.}}{\gamma }$ is the shear rate (s^−1^), *K* is the consistency coefficient (Pa·s*^n^*), and *n* is the flow behavior index (dimensionless) that signifies the extent of deviation from Newtonian behavior [27].

### 2.7. Experimental Design

Experiments were conducted following a single-factor completely randomized design (CRD). This factor was set as wheat/ackee aril flour mass ratio, following the gradual replacement of wheat flour by ackee aril flour. Five different levels of blending were analyzed by using the wheat to ackee aril flour mass ratios of 100 : 0 (T1; pure wheat flour); 75 : 25 (T2); 50 : 50 (T3), 25 : 75 (T4), and 0 : 100 (T5; pure ackee aril flour). The experiments were repeated three times, for a total of 18 experimental units. The response variables were pasting and rheological parameters.

### 2.8. Statistical Analysis

Means and standard deviations were calculated for all data. Analysis of variance (ANOVA) and Tukey tests at a significance level of 5 % were used to determine the influence of blending ackee aril flour with wheat flour on pasting and dough rheological properties. Data processing was carried out by using STATGRAPHICS® Centurion XVI (StatPoint Technologies, United States), while curve fitting was done through Origin® 7.5 (Origin Lab Corporation, United States).

## 3. Results and Discussion

This research focused on the evaluation of pasting and rheological properties of wheat flour (T1), ackee aril flour (T5), and wheat-ackee aril flour blends (T2, T3 and T4), with the gradual wheat flour replacement by ackee aril flour. The first part of this section was devoted to the analysis of the physicochemical composition of pure flours and their mixtures. Afterwards, an evaluation of pasting and dough rheological properties was conducted and related to the previous physicochemical results. Pasting temperature, peak viscosity, ease of cooking, swelling power, final viscosity at 50 °C, and thixotropy were analyzed when the pasting properties were studied. Then, steady-shear measurements were used to obtain and analyze the consistency coefficients (*K*) and flow behavior index (*n*) parameters obtained after the data were fitted to the Power Law and Herschel-Bulkley models. Yield stress parameters (*τ*_0_) were also obtained after data fitting by using the Herschel-Bulkley model.

### 3.1. Physicochemical Properties of Different Wheat Flours

Table 1 shows the composition profile of wheat flour, ackee aril flour, and their mixtures. Values with different lowercase letters within the same row in Table 1 had statistically significant differences (*p* < 0.05). As can be seen in Table 1, the gradual addition of ackee aril flour to the composite flour produced changes in the physicochemical composition of the obtained flour blend. The continuous increase in the ackee aril flour fraction produced a consecutive increase in ash, fat, protein, and fiber content, while water and carbohydrate content showed the opposite behavior. According to previous reports involving ackee aril flour [18, 22], the gradual changes in the composition of the obtained blends were due to the remarkably high protein content of the ackee aril flour.

Additionally, statistically significant differences (*p* < 0.05) were found among all formulations regarding moisture and ash content. In terms of fat and carbohydrate content, only T4 and T5 had no significant differences (*p* > 0.05). This lack of significant differences (*p* > 0.05) was also observed in pairs T1-T2 and T3-T4 in terms of protein and fiber content.

In general, a quasilinear relationship between the macronutrient content and mass ratio of flours was observed. Thus, ackee aril flour had a much higher protein content compared to pure wheat flour. The protein content of the flour without ackee aril had a protein content of 12 %; while increases of 25 %, 50 %, 75 %, and total replacement of 100 % of ackee aril flour resulted in flours with a protein content of 13 %, 16 %, 22 %, and 23 %, respectively, demonstrating the undoubted protein contribution of the ackee aril flour. As can be seen in Table 1, there was an almost linear relationship between the ackee aril flour fraction and the protein content of the final composite flour obtained. At this point, it should be pointed out that the high protein content found in ackee aril flour represents a nutritional opportunity for this raw material as a cheap and available source of protein in developing countries such as Colombia and most specifically in the Caribbean region.

### 3.2. Pasting Properties

Pasting properties of composite flours made by blending wheat flour with ackee aril flour in different mass ratios (100 : 0, 75 : 25, 50 : 50, 25 : 75, and 0 : 100) are shown in Table 2. Results indicated that an increase of ackee aril flour fraction progressively decreased the pasting parameters. This behavior was mostly related to the gradual increase of the protein content (and the decrease of carbohydrate content) in the analyzed doughs [28]. However, the statistical analysis showed significant effects of the blending level on only some pasting parameters. Thus, as can be seen in Table 2, the ackee aril flour proportion in dough did not significantly influence (*p* > 0.05) pasting temperature. Significant differences (*p* < 0.05) were only observed when pure ackee aril flour (T5) was analyzed. Pasting temperature values found in this work were similar to those reported for cassava dough [29]. Pasting temperatures found in this study could make it feasible to include these flours in products subjected to high processing temperatures, such as canned products [30], or even for the production of pasta [20].

T1 (pure wheat flour) and T2 (25 % by mass of ackee aril flour) had the highest peak and final viscosities at 50 °C. These parameters were directly affected by the higher carbohydrate content in wheat flour-rich doughs and the resultant swelling of the starch granules present [28]. The friction between the swollen granules and the water absorption competition between the starch and nongelatinized granules also influenced [28] the swelling power. These phenomena were also reflected in the highest swelling power of T1 and T2.

Regarding the aforementioned, the blend level significantly influenced (*p* < 0.05) the swelling power. However, at ackee aril flour mass fractions above 75 % (T4 and T5), there were no significant differences in swelling power (*p* > 0.05). The ackee aril flour content may have interfered with the adhesive interactions between the starch granules, resulting in lower adhesive forces that made water penetration difficult and thus lowering swelling power [5]. On the other hand, high values of thixotropy were found, indicating high viscoelasticity and a high degree of recovery of the internal dough structure [31]. Finally, high values of ease of cooking were found in doughs with a high ackee aril flour fraction; the highest value being found in the dough containing 75 % by mass of ackee aril flour (T4).

### 3.3. Rheological Behavior of Dough

**Figure 1:**
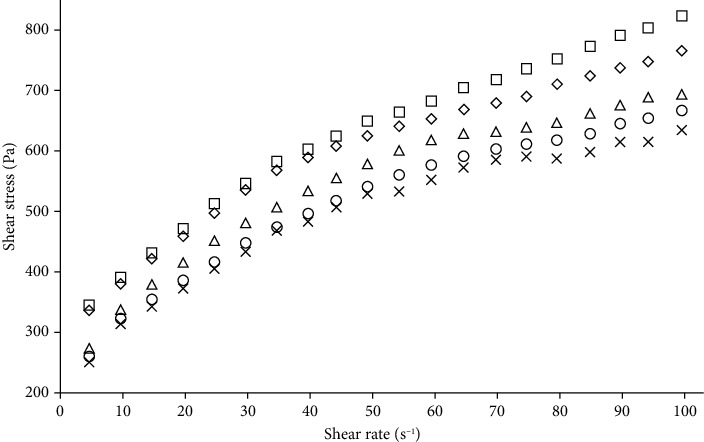
Shear stress (*τ*) and shear rate (*γ*) changes at 25°C for doughs with wheat to ackee aril flour mass ratios of 100 : 00 (T1, □); 75 : 25 (T2, ◊), 50 : 50 (T3, ∆), 25 : 75 (T4, O), and 0 : 100 (T5, х).

Figure 1 shows the shear stress and shear rate for dough with wheat to ackee aril flour mass ratios of 100 : 00 (T1), 75 : 25 (T2), 50 : 50 (T3), 25 : 75 (T4), and 0 : 100 (T5). As can be seen in Figure 1, all curves began at the origin of the shear stress-shear rate plot but were concave upwards; that is, an increase in the shear rate gave a less than proportional increase in shear stress. This was observed in all cases regardless of the initial formulation. The aforementioned is a classic behavior of shear thinning or pseudoplastic fluids. Shear thinning can be thought to be due to a breakdown of structural units in foods because of hydrodynamic forces generated during shearing. In this particular case, this phenomenon was related to the fact that the network formed by starch granules of wheat flour dough containing ackee aril flour was less adhesive during gelation, which resulted in the disintegration of the long-chain polymers, allowing easier breakage under the applied stress; therefore, the flow resistance decreased [27].

After the steady-shear measurements carried out in this work, consistency coefficients (*K*), flow behavior index (*n*), and yield stress (*τ*_0_) parameters were obtained after the data were fitted to the Power Law and Herschel-Bulkley models. These results are shown in Table 3. As can be seen in Table 3, the Power Law model was the one that best described the dough flow behaviors, considering that with this model, the highest regression coefficients (*R*^2^) were found.

It is also observed in Table 3 that the values of *n*, which reflect the flow behavior, were less than 1, thus supporting the pseudoplastic behavior of all samples. Furthermore, the gradual addition of ackee aril flour significantly increased (*p* < 0.05) *n* values in T3, T4, and T5 (doughs with 50 %, 75 %, and 100 % ackee aril flour, respectively) when evaluated by the Power Law model. The above suggested a decrease in the pseudoplastic behavior (decrease in shear thinning). However, these treatments did not present significant differences among them (*p* > 0.05). For the data evaluated by the Herschel-Bulkley model and regarding *n* values, the flow behavior was similar and there were no significant differences among treatments with wheat flour content higher than 25 %. A decrease of shear thinning with the addition of ackee flour was observed earlier during the pasting experiments.

On the other hand, the consistency index (*K*) of doughs evaluated by the Power Law model decreased with the higher presence of ackee aril flour, with statistically significant differences (*p* < 0.05) up to treatments with 75 % of ackee aril flour. This suggested the dependence of pseudoplasticity on the content of ackee aril flour. The highest consistency index was obtained for T1 (dough with 100 % wheat flour), while the lowest consistency index was obtained for T4 and T5 (dough with 75 % and 100 % ackee flour, respectively). The previous treatments decreased their consistency index by almost 30 % compared to T1. In general, these results suggested that starch granule interactions depended on the amylase/amylopectin ratio, which determines the strength and behavior of starch gels [32]. When the data were fitted with the Herschel-Bulkley model, the consistency index trend was similar to the case described above with the Power Law model, but significant reduction (*p* < 0.05) was only found when T5 was analyzed. In addition, an increase in the yield stress (*τ*_0_) was observed with the content of ackee aril flour in doughs, which reflected the presence of some cross-linked structures during gelatinization and cooling [27, 32].

These differences found in the rheological behavior of different dough composites were related to the variation in protein content and composition in these flours. In another similar study, Katyal et al. [28] evaluated the dough rheological properties of composite flours made from flour varied in gluten strength. Authors mixed strong wheat flour with very weak, weak, and medium wheat flours. Five different levels of blending were obtained with the mass ratios of strong flour: very weak/weak/medium flour (100 : 0, 75 : 25, 50 : 50, 25 : 75, and 0 : 100). The authors claimed that the protein content of the composite flour progressively decreased with a decrease in the proportion of strong wheat flour, which consequently changed dough rheological properties. Moreover, Katyal et al. [28] stated that the difference in rheological behavior of strong wheat flour with the blending of very weak, weak, and medium flours may be due to variation in protein content and composition in these flours, such as those found in this work.

## 4. Conclusions

The gradual replacement of wheat flour by ackee aril flour affected pasting properties and dough rheology. This phenomenon was associated with the almost linear change in flour composition with the ackee aril flour increase, mostly related to the protein and carbohydrate content. Moreover, doughs obtained from the gradual wheat flour substitution with ackee aril flour showed a pseudoplastic behavior with thixotropy whose viscosity decreased as the addition of ackee aril flour was increased.

Depending on the degree of blending, flour composites with different pasting and rheological properties, suitable for different pastry products, could be tuned by modifying the ackee aril fraction in the composite flour. These flours obtained by adding ackee aril flour had promising nutritional profiles, which could represent a cheap and available source of nutrients for low-income populations, such as the one in the Colombian Caribbean region, one of the most affected regions by poverty and armed conflict. Thus, the pasting properties found for ackee-wheat flour blends involving ackee aril flour from 25 % to 75 % of the obtained blends in this work indicate the possibility of using these flours in products subjected to high processing temperatures, such as canned products, or even to produce chips and pasta.

These results represent a breakthrough in the development of wheat flour substitutes, which is indeed a current challenge for Food Science. In this regard, and considering the physicochemical, pasting, and rheological properties of the doughs evaluated in this work, the obtained ackee aril flour has a considerable potential of use that should be further investigated in terms of optimal wheat flour replacement ratio for its possible application in the food industry for particular formulations and goods such as (but not limited to) canned goods, pasta, baked goods that require high temperatures, soups, sauces, sausages, jellies, children's food, and other products of the bakery lines.

## Figures and Tables

**Table 1:** Physicochemical properties of wheat flour (T1), ackee aril flour (T5), and blends with wheat to ackee aril flour mass ratios of 75 : 25 (T2), 50 : 50 (T3), and 25 : 75 (T4).

Physicochemical properties	Treatment (levels of blending: wheat to ackee aril flour mass ratio)				
	T1 (100 : 0)	T2 (75 : 25)	T3 (50 : 50)	T4 (25 : 75)	T5 (0 : 100)
Moisture (%)	13.4 ± 0.3^a^	11.3 ± 0.2^b^	9.2 ± 0.3^c^	7.6 ± 0.2^d^	7.3 ± 1.0^e^
Ash (%)	0.5 ± 0.0^a^	1.6 ± 0.1^b^	2.8 ± 0.0^c^	3.5 ± 0.2^d^	4.1 ± 0.0^e^
Fat (%)	1.7 ± 0.1^a^	8.0 ± 0.1^b^	14.2 ± 0.4^c^	20.5 ± 0.5^d^	21.4 ± 0.1^d^
Protein, *N* × 6.25 (%)	11.9 ± 0.2^a^	12.8 ± 0.1^a^	16.1 ± 0.3^b^	21.7 ± 0.8^c^	23.0 ± 0.2^c^
Fiber (%)	2.4 ± 0.0^a^	2.5 ± 0.0^a^	3.6 ± 0.1^b^	3.9 ± 0.2^c^	4.1 ± 0.3^c^
Carbohydrates (%)	72.5 ± 2.9^a^	59.2 ± 1.6^b^	49.3 ± 1.1^c^	44.0 ± 2.2^d^	42.5 ± 1.2^d^

Values are presented as mean ± standard deviation (*n* = 3 × 2). Different lowercase letters within the same row represent statistically significant differences at 5% significance.

**Table 2:** Effect of blending wheat and ackee aril flour on dough pasting properties.

Treatment	Pasting temperature (°C)	Peak viscosity (Pa·s)	Ease of cooking (min)	Swelling power (g water g starch^−1^)	Final viscosity (Pa·s)	Thixotropy (%)
T1	68 ± 0^a^	2451 ± 34^a^	1.4 ± 0.2^a^	1086 ± 14^a^	1171 ± 35^a^	27 ± 2^a^
T2	69 ± 4^a^	733 ± 46^b^	1.4 ± 0.3^a^	268 ± 12^b^	414 ± 8^b^	23 ± 1^b^
T3	67 ± 5^a^	292 ± 30^c^	1.6 ± 0.8^a^	56 ± 5^c^	204 ± 10^c^	19 ± 0^c^
T4	68 ± 2^a^	143 ± 23^d^	2.3 ± 0.8^b^	65 ± 3^d^	93 ± 6^d^	21 ± 0^c^
T5	50 ± 0^b^	110 ± 1^d^	1.4 ± 0.1^a^	75 ± 2^d^	71 ± 3^e^	19 ± 0^c^

Wheat/ackee aril flour mass ratios of 100 : 00 (T1), 75 : 25 (T2), 50 : 50 (T3), 25 : 75 (T4), and 0 : 100 (T5). The same lowercase letters within a column are not significantly different at *p* < 0.05.

**Table 3:** Flow parameters from the Power Law and Herschel-Bulkley models for wheat dough, ackee aril dough, and wheat-ackee aril dough mixtures.

Treatment	Power Law ($\tau =K{\dot{\gamma }}^{n}$)			Herschel-Bulkley model ($\tau =\tau_{0}+K{\dot{\gamma }}^{n}$)			
	*K* (Pa·s*^n^*)	*n*	*R* ^2^	*τ*_0_ (Pa)	*K* (Pa·s*^n^*)	*n*	*R* ^2^
T1	200 ± 5^a^	0.30 ± 0.00^a^	0.993	30 ± 5^a^	170 ± 10^a^	0.33 ± 0.00^a^	0.995
T2	190 ± 5^b^	0.30 ± 0.00^a^	0.993	65 ± 5^b^	150 ± 10^a^	0.33 ± 0.00^a^	0.995
T3	170 ± 5^c^	0.31 ± 0.00^b^	0.994	50 ± 6^c^	130 ± 10^a^	0.34 ± 0.04^a^	0.993
T4	155 ± 5^d^	0.31 ± 0.00^b^	0.996	40 ± 10^a^	120 ± 20^a^	0.35 ± 0.03^a^	0.986
T5	150 ± 5^d^	0.31 ± 0.00^b^	0.993	125 ± 10^d^	70 ± 10^b^	0.43 ± 0.00^b^	0.988

Wheat/ackee aril flour mass ratios of 100 : 00 (T1), 75 : 25 (T2), 50 : 50 (T3), 25 : 75 (T4), and 0 : 100 (T5). The same lowercase letters within a column are not significantly different at *p* < 0.05.

## Data Availability

The data used to support the findings of this study are available from the corresponding author upon request.
